# Ecdysterone and Turkesterone—Compounds with Prominent Potential in Sport and Healthy Nutrition

**DOI:** 10.3390/nu16091382

**Published:** 2024-05-02

**Authors:** Velislava Todorova, Stanislava Ivanova, Dzhevdet Chakarov, Krasimir Kraev, Kalin Ivanov

**Affiliations:** 1 Department of Pharmacognosy and Pharmaceutical Chemistry, Faculty of Pharmacy, Medical University-Plovdiv, 4002 Plovdiv, Bulgaria; 2 Research Institute, Medical University-Plovdiv, 4002 Plovdiv, Bulgaria; 3 Department of Propedeutics of Surgical Diseases, Section of General Surgery, Faculty of Medicine, Medical University-Plovdiv, 4002 Plovdiv, Bulgaria; 4 Department of Propedeutics of Internal Diseases, Medical Faculty, Medical University-Plovdiv, 4002 Plovdiv, Bulgaria

**Keywords:** 20-hydroxyecdysterone, turkesterone, phytoecdysteroids, *Rhaponticum carthamoides*, *Spinacia oleracea*, *Chenopodium quinoa*, *Ajuga turkestanica*

## Abstract

The naturally occurring compounds ecdysterone and turkesterone, which are present in plants, including *Rhaponticum carthamoides* Willd. (Iljin), *Spinacia oleracea* L., *Chenopodium quinoa* Willd., and *Ajuga turkestanica* (Regel) Briq, are widely recognized due to their possible advantages for both general health and athletic performance. The current review investigates the beneficial biological effects of ecdysterone and turkesterone in nutrition, highlighting their roles not only in enhancing athletic performance but also in the management of various health problems. Plant-based diets, associated with various health benefits and environmental sustainability, often include sources rich in phytoecdysteroids. However, the therapeutic potential of phytoecdysteroid-rich extracts extends beyond sports nutrition, with promising applications in treating chronic fatigue, cardiovascular diseases, and neurodegenerative disorders.

## 1. Introduction

Ecdysteroids are a class of invertebrate steroid hormones, first found in insects, in which they regulate activities such as molting, development, and reproduction, including the critical metamorphic phases in arthropods [1,2]. The first ecdysteroid, ecdysone, was isolated from silkworm pupae by Butenandt and Karlson in 1954, and its structure was presented in 1965 by Huber and Hoppe [3,4]. Nowadays, over 550 ecdysteroids are known [5]. They possess a tetrahydroxylated four-ring structure, a cyclopentanoperhydrophenanthrene skeleton consisting of 27–30 carbon atoms with a *β*-side chain at C17, originating from cholesterol or alternative sterols [1,6]. 

Ecdysteroids are classified into three main groups based on their natural origin, including phytoecdysteroids (PEs), zooecdysteroids, and mycoecdysteroids [1]. Phytoecdysteroids are a class of bioactive molecules produced by plants as a defense against herbivorous insects [7]. They are widely distributed through the plant kingdom, and research results suggest that only around 6% of plant species contain detectable levels of PEs [6,8,9,10]. Phytoecdysteroids were first reported in the mid-1960s and discovered in diverse plant sources, including the leaves of *Podocarpus nakaii*, the pinnae of *Pteridium aquilinum,* the bark of *Podocarpus elatus*, the roots of *Achyranthes fauriei*, etc. [11]. Some ecdysteroids, including ecdysone, 20-hydroxyecdysone (20HE), makisterone A, and ajugasterone C, are found in both botanical and zoological environments [6,8]. Phytoecdysteroids occur in a variety of plant families, including Asteraceae, Amaranthaceae, Commelinaceae, Liliaceae, Lamiaceae, Magnoliaceae, Podocarpaceae, Ranunculaceae, etc. Representative species of plant families include *Ajuga turkestanica* (Regel) Briq. (*A. turkestanica*), *Rhaponticum carthamoides* Willd. (Iljin) (*R. carthamoides*), *Pfaffia glomerata* (Spreng.) Pedersen, *Cyanotis arachnoidea* C.B. Clarke (*C. arachnoidea*), etc. [1,5,12]. These plants have been used as adaptogens, antioxidants, tonifying agents, and to promote muscle growth and strength since ancient times [13,14,15]. The most common and isolated PEs from these plants are 20HE, ajugasterone C, turkesterone, polypodine B, ponasterones A, B, and C, etc. [16,17]. Some of the essential factors for ecdysteroid levels in plants are the developmental stage, the growing conditions, and the ecotype [18]. It is considered that 20HE does not bind to the androgen receptor, suggesting that ecdysteroids may exhibit anabolic effects via a mechanism other than androgens. It has been proposed that they activate the PI3K pathway [19]. Dietary supplements (DSs), as well as dietary regimes and plant-based diets, have grown in popularity in recent years for a variety of reasons, including popular objectives, such as extending lifespan, improving health quality, facilitating weight loss efforts, and improving athletic performance [20,21,22,23,24,25]. In general, DSs are an important part of athletes’ dietary plans. These products not only enrich the diet but also have an essential role in performance, adaptation, endurance, and recovery [26,27]. With regard to the DSs intended to increase physical strength, some contain ecdysteroids due to their anabolic effects, adaptogenic potential, and ergogenic properties [23]. In general, the ecdysteroids usually found in DSs are 20HE and turkesterone [28]. Moreover, utilizing naturally derived steroids for enhancing muscle performance is preferred over using anabolic steroids [29,30]. Notably, 20HE is widely available in large amounts at competitive market costs and can be easily isolated and purified from selected plant species recognized for high accumulation rates [31]. Furthermore, the inclusion of 20HE in World Anti-Doping Agency (WADA) monitoring programs since 2020 demonstrates the increased regulatory attention given to these substances [15,32,33]. The inclusion of 20HE in the WADA monitoring program could be regarded as controversial because the compound also presents in some common foods, such as spinach (*Spinacia oleracea* L., Chenopodiaceae) and quinoa (*Chenopodium quinoa* Willd., Chenopodiaceae). These plant species are considered functional foods with good nutrition profiles [34,35,36,37] and are included in the nutritional regimens of many professional athletes, plant-based diets, and other healthy regimens. 

Plant-based diets not only gained popularity in the last several years but are also associated with a reduction in the risk of chronic diseases, including diabetes, heart disease, and certain cancers. These diets are typically higher in fiber, vitamins, minerals, and antioxidants [38]. Additionally, it is considered that plant-based diets have a smaller environmental footprint compared to diets high in animal products. Moreover, they often include specific foods, such as spinach quinoa [39]. The most common sources of ecdysterone and turkesterone in DSs are plants such as *C. arachnoides*, *A. turkestanica*, and *R. carthamoides* [28,34]. 

The purpose of this review is to assess the evidence for the beneficial role of ecdysteroids in human nutrition and indicate prospects for future research.

## 2. Materials and Methods

**Figure 1:**
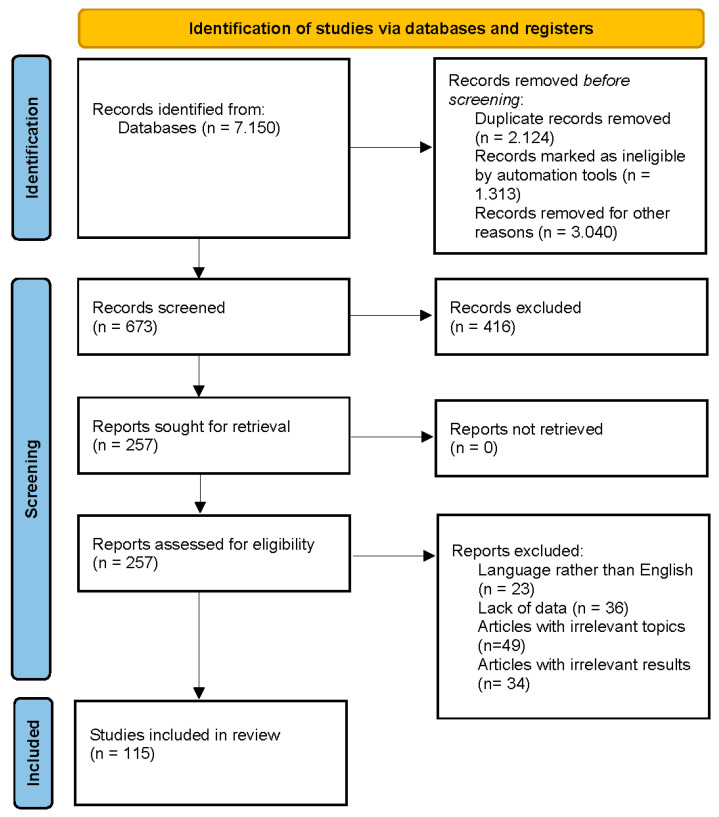
PRISMA 2020 flow diagram [40].

The search strategy was to investigate the biological activities of ecdysterone, turkesterone, *R. carthamoides*, *S. oleracea*, *C. quinoa*, and *A. turkestanica*. We screened original published research papers in databases such as Scopus, PubMed, Science Direct, and Google Scholar. We used a specific set of keywords and combinations to search the databases for research papers. These keywords and combinations were “ecdysterone”, “turkesterone”, “*R. carthamoides*”, “spinach”, “*Spinacia oleacea*”, “quinoa”, “*Chenopodium quinoa*”, “*Ajuga turkestanica*”, “biological activity”, and “biological effects”. We did not select a time frame for our search. The final step involved reading and identifying the selected articles. A total of 227 papers, of which 115 are about the biological activities of ecdysterone, turkesterone, and plants containing these PEs, were selected and included in the current review. This was carried out in accordance with the Preferred Reporting Items for Systematic Reviews and Meta-Analyses (PRISMA) criteria shown in Figure 1.

## 3. Results and Discussion

### 3.1. 20-Hydroxyecdysterone

Among more than 520 known ecdysteroids, one of the most common is 20HE, with various biological activities and commercially available substances. Ecdysterone is a structurally characteristic ecdysteroid—2β,3β,14α,20β,22α,25β-Hexahydroxycholest-7-en-6-one (ecdysterone, β-ecdysone, 20-hydroxyecdysone) [5,41]. Important for the biological activity of ecdysteroids are the double bond at C-7, the keto group at C-6, and the hydroxyl groups at positions C-2, C-3, C-14, and C-22, and the hydroxyl group at C-20 correlates with anabolic activity [28].

The role of 20-HE as a defensive chemical in plants has been well-established [18]. Although *R. carthamoides* is considered as one of the main sources of the compound, many other plant species that are rich sources of 20HE are *C. arachnoidea, Pfaffia* (*P. glomerata*, *P. iresinoides*), and Serratula (*S. centauroides*, *S. coronata*, etc.) [31]. It has been reported that *Serratula coronata* L. juice and the roots of *R. carthamoides* contain roughly 1.5% 20HE. The *C. arachnoidea* roots contained up to 4–5% 20HE. Ecdysteroids are also found in substantial amounts in agricultural products, such as spinach, sugar beets, and saltbush seeds. Ecdysterone is used as an ingredient in dietary supplements in the field of sports nutrition [12,17,26,34]. 

The precise mechanism of action of 20HE remains unclear, but it is hypothesized that it exerts its effects by enhancing protein synthesis in skeletal muscles and the heart [42], boosting ATP synthesis in muscles [43], reducing hyperglycemia in diabetic animals [44], lowering plasma cholesterol levels [45], promoting the production of red blood cells, and decreasing the activity of triglyceride lipase [31]. 

In recent decades, significant research has been performed on the potential performance-enhancing benefits and therapeutic applications of ecdysterone [15,23,28]. The World Anti-Doping Agency (WADA) [33] agreed to add ecdysterone to their monitoring program in 2020, and the inquiry has continued ever since. Moreover, ecdysterone is widely thought to be nontoxic to mammals. According to Ogawa et al., the LD_50_ values for orally administrated 20HE in mice exceeded 9 g/kg, whereas intraperitoneally injected 20HE had an LD_50_ value of 7.8 g/kg [46]. Seidlova-Wuttke et al. conducted a study in which ovariectomized rats were fed dosages of up to 500 mg/kg daily for three months with no obvious adverse responses [47]. In general, ecdysteroids are considered safe for mammals [46]. Nowadays, numerous DSs containing ecdysteroids are available on the market. The focus of most of these products is sporting individuals [48].

Faster adaptation, the stimulation of protein synthesis [49], stress and anxiety reduction [50], antioxidant defense, the protection of joint cartilage [51], and neuroprotection [52] are some of the most important benefits that foods and plants rich in 20-hydroxyecdysterone might provide. Table 1 presents in vitro studies investigating the biological effects of 20HE.

Table 1 provides a summary of studies on ecdysterone and various cellular processes and disorders. The data suggest that ecdysterone’s biological effects are associated with histamine release inhibition [54] and neuroprotective effects [55] and show promising therapeutic potential in Alzheimer's disease [55]. Ecdysterone possesses an anti-adipogenic effect, which may be important for future studies on ecdysterone in obesity treatment [69]. Moreover, the effects on cell lines are associated with endothelial dysfunction prevention, osteoporosis prevention, glucose regulation, cytotoxic effects, antibacterial properties, immunomodulatory effects, and anti-inflammatory activities [52,53,54,55,56,57,58,60,61,62,63,64,65,66,67,68,69,70,71,72,73]. Within these studies, no serious effects were reported [52,53,54,55,56,57,58,60,61,62,63,64,65,66,67,68,69,70,71,72,73]. 20-hydroxyecdysone has various pharmacological effects, demonstrating its potential as a therapeutic agent in numerous states, including allergy responses, neurological illnesses, cancer, and inflammatory disorders. However, in vitro studies are insufficient, and more in vivo studies are needed to determine the specific mechanisms of action and investigate their therapeutic uses. Table 2 presents in vivo studies and trials including 20HE.

The in vivo studies presented in Table 2, suggest that ecdysterone possesses effects on osteoblast differentiation and bone regeneration, joint morphology and osteoporosis, Alzheimer’s disease, and lipid metabolism, as well as having anti-obesity, anti-diabetic, and neuroprotective effects [47,49,50,51,74,75,76,78,79,80,81,82,83,84,85,86,87,88,89,90,91,92,93,94,95,96,97,98,99,100,101]. Some of the in vivo studies confirm the in vitro studies about anti-obesity, anti-diabetic, neuroprotective, and cytotoxic effects, as well as the prevention of Alzheimer’s disease. A controlled randomized study investigated the utilization of 20HE in metabolic syndrome [99]. Ecdysterone has great potential for use in medications intended to cure a variety of illnesses. Furthermore, ecdysterone does not appear to possess any severe side effects [19,47,49,50,51,74,75,76,77,78,79,80,81,82,83,84,85,86,87,88,89,90,91,92,93,94,95,96,97,98,99,100,101]. These suggest that ecdysterone supplementation is safe. In a study involving 20-hydroxyecdysone in the dietary supplement “Peak Ecdysone”, conducted on 46 men over a 10-week period, it was discovered that 20HE supplementation resulted in increases in body weight and muscle mass, as well as improvements in power and strength performance, without adverse effects or changes to the steroid profile [26]. It is considered that lower doses showed no significant effects but reported that amounts more than 5 μg/kg body weight were considered effective [102]. This shows that ecdysterone administration may be beneficial for improving athletic performance without compromising health. 

We found multicenter randomized double-blind studies on ecdysterone and ecdysterone-rich extracts. Further research is needed to understand the mechanisms of action and possible long-term consequences of these supplements. To fully assess the benefits and possible future applications of ecdysterone, multicenter randomized double-blind trials are required. 

### 3.2. Rhaponticum carthamoides

*Rhaponticum carthamoides* (Willd.) Iljin is a perennial herb of the Asteraceae family that is also known as maral root or Russian leuzea. It grows in the harsh conditions of South Siberia’s Altai and Saian mountains. It is a semi-rosulate plant that may grow to be 150 cm tall [13,103,104]. The use of *R. carthamoides* for medicinal purposes dates back to ancient times, and traditional Siberian medicine has long praised the plant for its ability to treat weariness and debility after sickness [13,103]. In the history of Russian scientific investigation, *R. carthamoides* has received a lot of attention in the domain of physical performance improvement. Research over the last century has shown its muscle- and strength-building capabilities, resulting in widespread use among elite athletes in Soviet and Russian sports [13]. In 1969, Brekhman and Dardymov classified *R. carthamoides* as an adaptogen, now widely used in herbal medicine to promote resistance to stress, such as trauma, anxiety, and fatigue [13,103,105]. 

**Figure 2:**
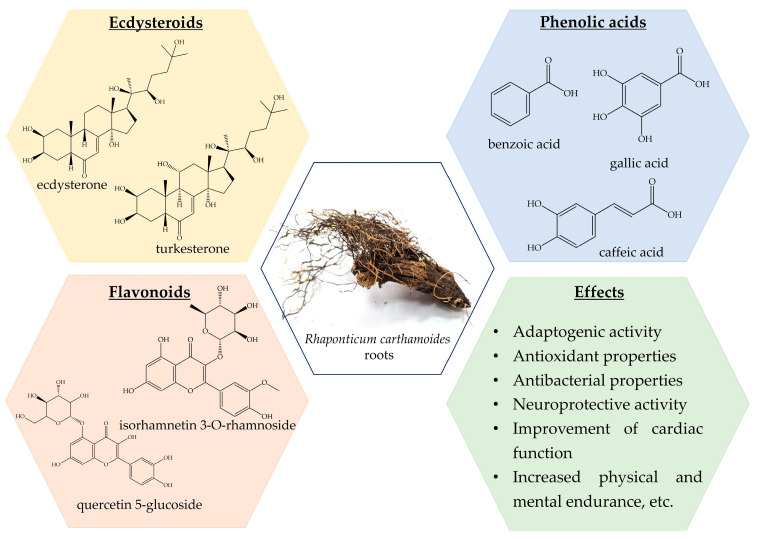
*Rhaponticum carthamoides* phytochemical composition and effects.

Previously, a wide range of chemical classes was found in *R. carthamoides* roots, with steroids, particularly ecdysteroids, flavonoids, lignans, and phenolic compounds (Figure 2) [13,106,107,108,109,110,111,112,113]. Moreover, *R. carthamoides* roots are a source of essential oil, which is characterized by antimicrobial, antioxidant, and anti-inflammatory effects [13,107,114,115,116,117]. Fifty different ecdysteroid chemicals have been found in the plant’s roots, aerial parts, and seeds [13,113]. An examination into the PE composition of *R. carthamoides* revealed the extraction of 20HE, inokosterone, leuzeasterone, polypodine B, rhapisterone, makisterone, carthamoleusterone, turkesteron, and their derivates from its underground parts [13,115,118]. The concentration of 20HE (β-ecdysone, ecdysterone, and polypodine A) varies between 0.049% and 1.74% [107,112,119]. 

Extracts from its roots and rhizomes are currently employed in a wide range of DSs and nutraceutical formulations. They are used to increase muscle growth, alleviate impotence, and reduce physical and mental fatigue [13]. Furthermore, *R. carthamoides* and its derivatives are used in cosmetics and herbal teas [13,106]. *Rhaponticum carthamoides* gained serious popularity in the last decade, especially after the introduction of numerous DSs containing leuzea extracts [120]. 

One of the most important benefits of leuzea supplementation is the potential to increase the working capacity of the skeletal muscles [42]. However, for the athletes, the adaptogenic activity of leuzea seems to be of the greatest importance [103]. Table 3 summarizes a wide range of in vivo investigations on the pharmacological activities of *R. carthamoides*.

Extracts from *R. carthamoides* show a variety of activities, including antibacterial, cytotoxic, anti-adipogenic, and antioxidant [69,121,122,123,124,125,126,127,128,129,130,131,132]. The investigations into *R. carthamoides* revealed that its extracts are low in toxicity [69,121,122,123,124,125,126,127,128,129,130,131,132]. In vitro research on cell cultures may not give comprehensive answers, but they serve as a basis for future in vivo investigations. Table 4 summarizes the findings from in vivo research and trials with *R. carthamoides* extracts.

The diverse range of bioactivities of *R. carthamoides* are presented in Table 4. These investigations include a wide range of activities, such as cognitive function effects, metabolic regulation, stress management, and cytotoxicity [44,133,134,135,136,137,138,139,140,141,142]. Mosharrof et al. investigated the effect of *R. carthamoides* extract on learning and memory processes in rats [133]. Petkov et al. found that an aqueous-alcoholic extract of *R. carthamoides* exhibited significant stimulating effects on the central nervous system, including improved learning and memory ability in rats [134]. Dushkin et al. investigated the therapeutic potential of *R. carthamoides* extract, as well as extracts from *Glycyrrhiza glabra* and *Punica granatum*. They observed that treatment with *R. carthamoides* extract significantly improved glucose and lipid metabolism compared to the other extracts [135]. Furthermore, studies on the combination administration of *R. carthamoides* and *Rhodiola rosea* on resistance exercise, the combination of these extracts boosted muscle protein synthesis and average power performance in rats [136]. These findings provide valuable insights into *R. carthamoides’* therapeutic potential for enhancing overall health and well-being. There are no observable indications or symptoms of toxicity, indicating a wide margin of safety [44,133,134,135,136,137,138,139,140,141,142]. Contrary to the randomized trial, which reported improved physical performance after the intake of 20HE, the investigation into the effects of *R. carthamoides* and *R. rosea* extracts suggests no potential effects on physical performance and fatigue [26,142]. These made *R. carthamoides* a valuable resource in sports nutrition. Further research is needed to understand the mechanisms of action and possible long-term consequences of these supplements. Moreover, the multicenter randomized controlled trials are limited.

### 3.3. Spinacia oleracea

Spinach (*Spinacia oleracea* L.) is an annual plant belonging to the family Chenopodiaceae; it can be divided into two subspecies, ssp. *glabra* and ssp. *spinosa* [143,144]. It originated in central Asia, specifically Persia, and its use dates back to ancient times [145,146]. 

**Figure 3:**
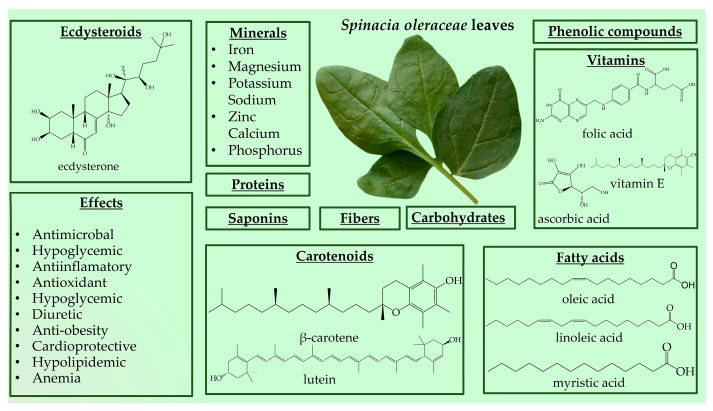
*Spinacia oleracea* nutrient profile and biological effects.

Spinach is a great source of nutrition and phytochemical constituents [143,144,146,147]. The nutrient composition can be divided into six major components, including carbohydrates (approximately 50%), proteins (approximately 14%), fats (approximately 23%), fiber, minerals, and vitamins (Figure 3) [146,148,149,150,151,152]. The high iron content makes it a valuable food for anemia [143]. A disadvantage of spinach is its high nitrate content, which may cause methemoglobinemia [143]. 

Spinach is source of beneficial phytonutrient constituents, such as phenolic compounds (flavonoids, phenolic acids, stilbenes, and lignans) and carotenoids (lutein, zeaxanthin, and *β*-carotene) [146,149,153]. Spinach also contains steroids, terpenes, tannins, and cardenolides [154]. Moreover, *Spinacia oleracea* produces large amounts of PEs, with the major component being 20HE [155,156]. Spinach has stimulated ecdysteroid accumulation in response to mechanical or insect injury, and PEs are metabolically stable in this plant species [11,157]. The reported content of 20HE in spinach leaves ranges from 17.1 to 885 µg/g [158,159]. However, variations exist regarding the levels of 20HE in spinach, with some reporting lower amounts, such as 50 μg/g dry mass, 10.3 and 16.8 μg/g in different spinach accessions, and 0.44 mg% dry weight [8,156,160]. Grucza et al. reported even lower levels of 20HE content in fresh spinach leaves at 10 µg/100 g [161]. These discrepancies in 20HE and phytochemical content may be attributed to different processing methods applied to spinach leaves [149].

Spinach is a versatile plant that may be consumed fresh in salads and smoothies or cooked in recipes such as steamed vegetables, casseroles, and soups [143,144,146,147,149]. Due to the wide variety of bioactive and phytochemical compounds, *S. oleracea* has a wide variety of potential functionalities, mainly antioxidant, antimicrobial, anticancer, anti-obesity, hypoglycemic, and hypolipidemic [146,149,151]. Spinach leaves are utilized for their, emollient, wholesome, antipyretic, diuretic, laxative, and anthelmintic substances, as well as their anti-inflammatory effects and joint pain relief (Figure 3) [151]. Spinach, as a significant antioxidant and nitric oxide donor, is a valuable vegetable in athletes’ diets [162]. In recent years, the consumption of spinach has increased due to consumers’ concerns about healthy eating [144]. Spinach’s traditional claim to increase muscle strength may have scientific support due to its ecdysteroid concentration. Because of its anabolic qualities, ecdysterone has gained popularity as a natural sports performance booster. As a result, ecdysterone-containing dietary supplements made from spinach and other plant extracts have become increasingly popular. Athletes are advised to take up to 1000 mg of ecdysterone daily; however, even high daily consumption of spinach (1 kg) rarely surpasses 100 mg. Studies on humans that have been given supplements containing ecdysterone have, over time, demonstrated increases in physical strength and muscular mass [34,163]. In Table 5 are presents in vivo studies on the biological activities of *S. oleracea*. 

Table 5 summarizes investigations into potential health benefits connected with *S. oleracea*. Gorelick-Feldman et al. investigated the effects of PEs and extracts from *A. turkestanica* and *S. oleracea* on protein synthesis and physical performance [19]. Panda et. al. investigated whether spinach extract may reduce hyperlipidemia by reducing pancreatic lipase activity. This suggests the importance of consuming spinach in managing lipid levels and obesity [164]. The spinach extract demonstrated promising appetite suppression effect which make it potential nutrient in appetite regulation [165].

These results show that spinach is associated with numerous health advantages, such as antioxidant characteristics, lipid-lowering effects, appetite regulation, tissue regeneration, and protection against oxidative stress-induced damage [156,162,164,165,166,167,168,169,170]. These highlight spinach’s potential as a useful nutrient. The data from in vivo experiments with animals are insufficient. Table 6 presents controlled trials involving *S. oleracea*.

The table presents randomized controlled trials investigating the effects of spinach and its derivatives. In a study on daily supplementation with *S. oleracea* extract, combined with moderate-intensity exercise, in over 50-year-old individuals, Perez-Pinero et. al. reported that spinach extract increases muscle-related factors and muscle quality [171]. Due to these results, spinach can be included in the nutritional diets of athletes, combined with physical exercises. Bohlooli et. al. reported that in well-trained men, daily oral supplementation with spinach reduced indicators of oxidative stress and muscle injury after training [162]. In a randomized control trial, Maruyama et. al. reported that the consumption of spinach in obese and insulin-resistant patients supplies antioxidants and improves lipid profiles in patients [172]. Also, a potential therapeutic application of spinach-derived thylakoid supplementation might be obesity management [174]. Moreover, the consumption of spinach extract significantly reduced hunger and increased postprandial plasma glucose concentrations, indicating a potential role in appetite regulation [175]. The findings from these studies collectively underscore the diverse health-promoting effects of spinach and its derivatives, ranging from muscle health and oxidative stress reduction to metabolic regulation [162,171,172,173,174,175,176,177,178]. Furthermore, the consumption of spinach is not associated with adverse effects [162,171,172,173,174,175,176,177,178]. These effects and the safety profile of spinach could correspond with its use in sports nutrition. Further double-blind multicenter randomized controlled trials are required to confirm the good therapeutic profile of spinach.

### 3.4. Chenopodium quinoa

Quinoa (*Chenopodium quinoa* Willd., Chenopodiaceae) is an annual herbaceous plant that is classified as a gluten-free pseudo-cereal [179,180]. Quinoa originated in the Andes of South America, specifically Peru, Bolivia, Ecuador, Colombia, and Chile [179,181]. The consumption of quinoa dates back 5000 years [182]. It grows to a height of 3–7 feet, the woody stem can be branched or unbranched, and it comes in different colors (green, red, and purple) [179,181]. Quinoa seeds are small, spherical, and flat, with colors that range from white to grey, black, yellow, and red [182,183].

**Figure 4:**
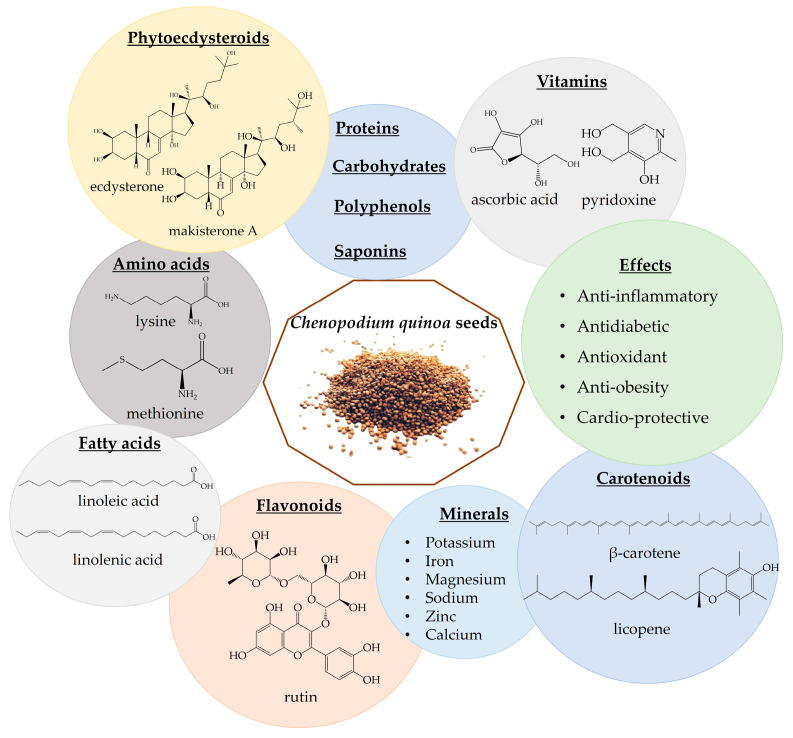
*Chenopodium quinoa* phytochemicals and biological effects.

The quinoa grain is characterized by high protein (13–15%), fiber (3–4%), carbohydrates (59–61%), fatty acids, vitamins, amino acids, and minerals [35,184,185,186,187,188]. Quinoa also contains a broad spectrum of polyphenols, carotenoids, phytoecdysteroids, phytosterols, saponins, and tannins [185,186]. There are about 36 different types of hytoecdysteroids detected in quinoa seeds, with high amounts of 20-hydroxyecdysone (up to 90% of PE content) and lower levels of makisterone A, 24(28)-dehydromakisterone A, 24-epi-makisterone A and polypodine B (Figure 4). It also contains PE derivates, such as 25,27-dehydroinokosterone, 24,25-dehydroinokosterone, and 5β-hydroxy-24(28)-dehydromakisterone A [12,35,189,190,191,192,193,194]. It is considered that both quinoa and spinach are poor sources of ecdysterone [36], compared to *R. carthamooides* and *A. turkestanica* [5,12,13]. However, quinoa seeds contain 4–12 times more 20HE by dry weight than spinach leaves [19,189,195].

Quinoa seeds have been utilized as flour, added to soups, and incorporated into bread recipes. The rise of new food products featuring ancient grains, including quinoa, is observed globally, offering new opportunities for these nutritious grains in the market [196,197]. Quinoa seeds are considered a “functional food”; it is considered that the consumption of 50 g of quinoa for 6 weeks is safe [198,199]. Apart from being rich in nutrients, quinoa also exhibits health-promoting properties, including anti-inflammatory, antidiabetic, antioxidant, anti-obesity, and cardio-beneficial effects (Figure 4) [35,191,192,193]. Moreover, not only the presence of phytoecdysteroids but also the presence of amino acids could increase muscle performance and lead to an increase in lean mass [200]. Table 7 presents in vitro studies involving quinoa.

The in vitro studies presented in Table 7 reported quinoa’s effects on gut microbiota and cancer cells, antioxidant and antidiabetic properties, platelet activity, and modulating collagenase activity [201,202,203,204,205,206,207]. Quinoa’s polysaccharides enhanced the synthesis of short-chain fatty acids and the composition of the microbiota. They might function as prebiotics [201]. Gawlik-Dziki et al. reported that quinoa possesses possible chemopreventive and anticarcinogenic qualities [202]. Furthermore, quinoa’s potential as a rich source of antioxidants is highlighted by the study by Alvarez-Jubete et. al. examining the polyphenol content and antioxidant qualities of methanolic extracts from the grain [203]. Overall, these studies highlight quinoa’s broad bioactive qualities and promise as a functional food. More studies, including in vivo, are required to explain the mechanism of action and the potential use of quinoa in DSs. Table 8 presents in vivo studies involving quinoa.

The studies presented in the table correspond with the potential effects of quinoa extract. Foucault et. al. conducted a study on the ability of quinoa extract enriched in 20HE to prevent obesity, and it was observed that quinoa and 20HE demonstrate anti-obesity activity in rats [208]. Moreover, Foucault et. al. reported that quinoa extract and 20HE regulated glucose and lipid metabolism [211]. Sidorova et. al. revealed that quinoa effectively normalized oxidative stress markers and improved cognitive function [209]. Meneguetti et al. reported that hydrolyzed quinoa decreased body weight, blood triacylglycerol level, and fat deposition [200]. Supplementation with quinoa and chia seed extracts may regulate metabolic indicators and showed hepatoprotective, anti-inflammatory, and antioxidant activities in rats [212]. Quinoa extracts showed anti-obesity, antioxidant, hypoglycemic, immunoregulatory, and collagenase-modulating effects in in vivo experiments with rats [208,209,210,211,212,213,214]. No serious adverse effects were reported within the experiments, which correlated with the safety profile of quinoa extracts and their safe nutrition in sports. No double-blind multicenter randomized controlled trials were found, so further trials are recommended to be conducted. 

### 3.5. Turkesterone and Ajuga turkestanica

Turkesterone is a PE, with 27 carbon atoms and seven OH-groups; it is considered that the OH groups at C-20 and C-11 are responsible for the anabolic effects [28,215]. Turkesterone is considered to possess an anabolic effect, and it is used as DS by athletes, instead of anabolic steroids [28,30]. It is found in some endemic plants, such as *A. turkestanica*, *R. carthamoides*, *Triticum aestivum* L., *Vitex scabra*, etc. [13,69,216,217,218]. The highest content of turkesterone is considered to be in *Ajuga turkestanica* [19]. Moreover, in DSs, turkesterone is derived from *A. turkestanica* [28].

Ecdysteroid-enriched preparations of *A. turkestanica* (or pure turkesterone) exhibit anabolic effects [216,219,220]. 

*Ajuga turkestanica*, a plant species of the Lamiaceae family, grows wild in the Boysun mountainous region of Central Asia, Uzbekistan. It is a perennial plant, that normally grows on clay, petrous, and rubbly slopes and rocks, with a height of 40–60 cm [14]. Both the aerial parts and root sections are utilized in folk medicine to prevent obesity, hair loss, and gastrointestinal diseases [14,216]. 

The phytochemical profile of *A. turkestanica* has indicated the existence of several biologically active secondary metabolites, such as carbohydrates, iridoids, diterpenes, phytoecdysteroids, flavonoids, sterol glycosides, and phenylethanoid glycosides [14,53,216]. The *A. turkestanica* extract contained 2.1% (*w*/*w*) turkesterone and 0.9% (*w*/*w*) 20HE [19]. Other isolated Pes are cyasterone, ajugasterone B, α-ecdysone, ecdysone 2,3-monoacetonide, and 22-acetylcyasterone [59,221,222]. 

Extracts from *A. turkestanica* are associated with wound-healing effects [223], as well as antiproliferative, anti-stress and immunostimulating, antimicrobial, and antioxidant effects [12,59]. Most of these effects are likely due to the presence of Pes [224]. Table 9 presents studies about the biological effects of turkesterone. *Ajuga turkestanica* and turkesterone products are commercially available not only online but also in bodybuilding centers [12]. Table 9 presents studies on the biological effects of turkesterone, while Table 10 presents studies on *A. turkestanica*.

The studies presented in the tables reported the various effects of turkesterone and *A. turkestanica*. Turkesterone is associated with a reduction in lipid accumulation in human adipocytes [69]. In a stress-induced mouse model, ecdysterone and turkesterone were found to prevent stress-related consequences and restore immunological activity [138]. Additionally, turkesterone exhibited beneficial effects on the endocrine and exocrine function in alloxan-induced diabetic rats, suggesting its potential therapeutic application in diabetes management [225]. Furthermore, PEs from *A. turkestanica* demonstrate hypoglycemic activity in models of hyperglycemia and diabetes, suggesting their potential therapeutic use in managing blood glucose levels [227]. Turkesterone presents a safer option to conventional anabolic steroids, displaying significant anabolic effects without inducing androgenic side effects in in vivo animal studies [19,219]. Moreover, *A. turkestanica* also enhances muscle regeneration and maintenance [29,226]. These results suggest that turkesterone extracted from *A. turkestanica* possesses diverse physiological effects, including potential performance enhancement and antiadipogenic, immunomodulatory, and hypoglycemic activities, making it a promising candidate for sports nutrition. Further research is needed to fully understand the mechanisms of action and therapeutic potential of turkesterone and *A. turkestanica* [19,29,59,69,77,138,219,220,225,226,227].

## 4. Conclusions

Phytoecdysteroids, widely distributed across various plant species, have been valued for their adaptogenic properties and potential to enhance muscle strength and performance. 20-hydroxyecdysone and turkesterone are two of the most noteworthy PEs, which are found in plants such as *R. carthamoides*, *S. oleracea*, *C. quinoa*, and *A. turkestanica*. Nowadays, these plants are used not just in plant-based diets for their high nutritional value but also as major components of DSs. Since 2020, ecdysterone has garnered attention and has been included in the WADA’s monitoring program due to its ability to enhance physical endurance. Studies on the benefits of 20HE, turkesterone, and extracts from these plants are limited. The preparations from these plants show promise not only as nutritional supplements but also as therapeutic agents for treating medicinal disorders, such as chronic tiredness, heart disorders, and neurodegenerative diseases. Future double-blind randomized multicenter trials are critical for fully assessing the effectiveness and safety of these preparations for cardiovascular disease, chronic tiredness, obesity, and osteoporosis. These studies will provide valuable insights into the therapeutic potential of phytoecdysteroid-rich extracts.

## Figures and Tables

**Table 1:** 20-hydroxyecdysone in vitro studies.

Study Objectives	Cell Lines	Main Results	Ref.
Neuroprotective effects of 20HE in Parkinson’s disease.	PC12 cells.	β-ecdysterone’s neuroprotective effects against MPPþ toxicity may be due to its antioxidant properties, which affect the PI3K-Nrf2 pathway.	[52]
Methanol, butanol, chloroform and water extracts and phytoecdysteroids (including ecdysterone) isolated from *Silene wallichiana* Klotsch.	Bacterial strains: *Klebsiella oxytoca* 6653, *K. pneumoniae* 40602, *K. aerogenes* NCTC8172, *Citrobacter freundii* 82073, *Staphylococcus aureus* MRSA16, *Enterococcus faecalis* NCTC775, *Proteus rettgeri* NCIMB9570, *Pseudomonas aeruginosa* NCTC6749, *Escherichia coli* NCTC9001, *Enterobacter hormaechei* T2, *Acinetobacter* sp. T132, *Pantoea agglomerans* T26, and *Bacillus cereus* T80. HeLa and HepG-2 cell lines.	At a MIC of 2.5 mg/mL, the methanolic extract inhibited the growth of *Acinetobacter* sp., *E. faecalis*, *K. oxytoca*, *P. agglomerans*, *P. rettgeri*, *P. aeruginosa*, and *S. aureus*, while *E. coli* and *K. pneumoniae* were inhibited at a MIC of 1.25 mg/mL. The chloroform extract significantly suppressed the growth of cancer cells. Aqueous and butanol extracts have high antioxidant activity.	[53]
20-hydroxyecdysone effects on histamine release.	Rat peritoneal mast cells.	20-hydroxyecdysone decreased the anti-IgE-induced histamine production by mast cells in a dose-dependent manner.	[54]
20-hydroxyecdysone protects SH-SY5Y cells from *β*-induced apoptosis.	Human neuroblastoma cells (SH-SY5Y).	20-hydroxyecdysone exerts neuroprotective effects through different and complementary pathways. It is a promising agent for the treatment of Alzheimer’s disease.	[55]
20-hydroxyecdysone	Human proximal tubular epithelial cells (HK-2).	20-hydroxyecdysone is a potential anti-fibrosis agent for renal proximal tubule cells.	[56]
Protective effects of 20-HE against endothelial dysfunction and its targets.	Human vascular endothelial cell line—HUVECs (CRL-1730 cells).	20-hydroxyecdysone inhibits inflammation via SIRT6-mediated NF-κB signaling in endothelial cells. 20-hydroxyecdysone could be a promising therapeutic avenue for addressing cardiovascular ailments.	[57]
Effect of glucocorticoids in osteoblasts and the role of 20HE in the pathogenesis of glucocorticoid-induced osteoporosis.	Bone marrow mesenchymal stem cells, isolated from C57BL/6 mice.	The protective effect of 20HE on dexamethasone-induced osteoporosis was also shown. It can induce osteogenic differentiation and autophagy.	[58]
*Ajuga turkestanica*, isolated compounds (including ecdysterone and turkesterone) antioxidant, cytotoxic, and antimicrobial activities.	HeLa (cervical cancer), HepG-2 (hepatic cancer), and MCF-7 (breast cancer) human cell lines. Growth of *Enterococcus VanB* VRE ATCC 902291, *E. VanB* VRE ATCC 31299, *S. aureus* MRSA ATCC 1000/93, and *S. aureus* MRSA ATCC 10442, *S. aureus* MRSA ATCC 10442, *S. pyogenes* ATCC 12344, *Candida albicans* ATCC 90028, *Candida glabrata* ATCC MYA 2950, *K. pneumonia* ATCC 700603, *P. aeruginosa* ATCC 27853.	The isolated ecdysteroids and iridoid glucosides from *A. turkestanica* were found to lack antioxidant properties and did not exhibit significant cytotoxic or antimicrobial effects. Interestingly, the chloroform extract, containing more lipophilic compounds, demonstrated higher activity.	[59]
Investigation of the potential of ecdysterone to lower glucose levels in hepatocytes and whether it stimulates insulin secretion.	HepG2 and βTC3 cell lines.	Ecdysterone may exert the glucose-lowering effect in hepatocytes that is insulin-independent and shows no effect on insulin release.	[60]
Effect of ecdysterone on human breast cancer cell lines.	MCF7, MDA-MB-231, MDAMB-468, DF2, and WI-38—breast cancer cell lines.	Tumor suppressive effect of 20HE on a panel of breast cancer cell lines. It shows synergism with doxorubicin to induce cell death in breast cancer cell lines.	[61]
Examine the effect of 20HE on the susceptibility of *Escherichia coli* to antibiotics.	*Escherihia coli* BW25113 (wild type) and *E. coli* strains NM3031 and NM3041.	In *E. coli*, 20HE altered antibiotic bactericidal effect.	[62]
20-hydroxyecdysone and its derivatives have the ability to sensitize multidrug-resistant (MDR) and non-MDR cancer cell lines to chemotherapeutic agents, and liposomal formulations.	MDR and non-MDR cancer cell.	20-hydroxyecdysone-diacetonide can sensitize both MDR and non-MDR cancer cell lines to chemotherapeutic agents, and liposomal formulations.	[63]
Effect of 20HE and ecdysteroid fraction isolated from *Serratula coronata* L. on human blood cells and neutrophils.	Erythrocyte rosette formation.	20-hydroxyecdysone exhibits potent immunomodulatory effects. It’s more effective than levamisole.	[64]
*Silene guntensis* B. Fedtsch and three isolated phytoecdysteroids incl. ecdysterone—antiproliferative and antioxidant effects.	Hepatocellular carcinoma (HepG-2), breast adenocarcinoma (MCF-7), and human cervix adenocarcinoma (HeLa) cell lines.	The chloroform extract showed potent cytotoxic effects, while the water and *n*-butanol extracts demonstrated notable antioxidant activities.	[65]
Investigation of 20HE, ajugasterone C, and polypodine B isolated from *Serratula coronata* effects on breast cancer.	Human breast cancer cells.	20-hydroxyecdysone and ajugasterone C demonstrated notable inhibition of viability in the triple-negative cell line. 20-hydroxyecdysone induction of proapoptotic activity in both MDA-MB-231 and T-47D cells.	[66]
Interaction between the ecdysterone isolated from *Sesuvium portulacastrum* and nitric oxide synthase.	3T3-L1 mouse fibroblast cells and RAW 264.7 mouse macrophage cells.	Ecdysterone boosts Nitric Oxide production and regulates intricate cellular mechanisms by activating ERK1/2 via the EGF pathway.	[67]
Cytotoxic effects of the butanolic extract from the underground parts of *Helleborus caucasicus* and the purified compounds: furostanol derivative, 20HE, and deglucohellebrin, on human cancer cell lines.	Calu-1—human epithelial lung cancer cells, MRC-5—human normal lung fibroblasts, and Caco-2—human colon cancer cells.	The extract of *H. caucasicus* and isolated compounds decreased cell viability in vitro on a lung cancer cell line and exerted a strong cytotoxic effect. Moreover, 20-HE and deglucohellebrin possess pro-apoptotic activities.	[68]
Antiadipogenic activity of *R. carthamoides* extract, ecdysterone, turkesterone, and ponasterone A.	SGBS human adipocytes.	*Rhaponticum carthamoides* extract, ecdysterone, and turkesterone reduced lipid accumulation in human adipocytes and *R. carthamoides* and ecdysterone stimulated lipolysis.	[69]
Effects of ecdysterone on interleukin-1β (IL-1β)-induced apoptosis and inflammation in rat chondrocytes.	Chondrocytes isolated from knee joints of 3 to 4-week-old male Sprague–Dawley rats.	Ecdysterone exhibited anti-apoptosis and anti-inflammatory effects in rat chondrocytes induced by IL-1β, which may be related to the NF-κB signaling pathway.	[70]
Effects of ecdysterone on brain acetylcholinesterase (AChE).	Rat cerebral cortex slices from Wistar albino rats.	Ecdysterone caused an increase in AChE in rat brain slices. No effect was observed in an in vitro assay using purified AChE.	[71]
Effects of β-ecdysone on osteoblast viability by assessing apoptosis following treatment with excess glucocorticoids.	BMSCs were isolated from the femurs and tibiae of mice—male BALB/C mice aged 8 weeks (20 ± 2 g body weight).	Beta-ecdysone prevented glucocorticoid-induced osteoblast apoptosis in vitro.	[72]
Anti-inflammatory activities of ecdysteroids from *Diploclisia glaucescens* (incl. ecdysterone).	Rat polymorphonuclear leukocytes (PMNs).	Tested compounds incl. ecdysterone showed significant anti-inflammatory activities.	[73]

**Table 2:** 20-hydroxyecdysterone in vivo studies and trials.

Study Objectives	Study Design	Main Results	Ref.
Mechanism of action of phytoecdysteroids (20-HE, polypodine B, and ponesterone) and *A. turkestanica* and *S. oleracea* in mammalian tissue.	A mouse skeletal muscle cell line, C2C12 (ATCC CRL-1772). Male Sprague–Dawley rats, divided into four groups: vehicle, 50 mg/kg 20HE, 1000 mg/kg spinach extract, or 10 mg/kg methandrostenolone given orally. Duration: 28 days.	In vitro, phytoecdysteroids increased protein synthesis by up to 20% in C2C12 murine and human primary myotubes. Improved grip strength in rats, according to in vivo research. Similarly, plant extracts had comparable results.	[19]
Metabolic effects of 20HE.	Sprague–Dawley rats. Five groups 12 rats/group: control—soy-free, pelleted food, estradiol group—10 mg/kg food, 20HE—1 g/kg food, 3 g/kg food and 6 g/kg food.	Ecdysterone has beneficial effects on fat and muscle tissue and may have a non-estrogenic mechanism for the prevention of metabolic syndrome and sarcopenia.	[47]
Ecdysteroids (including ecdysterone and turkesterone) isolated from *R. carthamoides*, *Silene brahuica Boiss*, *Silene praemixta* M. Pop., and *Ajuga turkestanika* (Rgl.)Brig.	Male rats. Phytoecdysteroids were administered perorally—single daily dose—5 mg/kg for 10 days. Reference group received methylandrostenediol or nerobol at a dose of 10 mg/kg.	Ecdysteroids are potential agents capable of stimulating protein synthesis in the body without affecting the endocrine system.	[49]
20-hydroxyecdysone-enriched fraction from *Pfaffia glomerata* roots.	125 Male C57BL/6J mice. Naive group (not stressed or treated mice). Second: stress-inducted mice without treatment. Tirth: stressed mice and treated with 3, 10, 30 mg/kg of 20HE-enriched fraction.	A 30 mg/kg dose of 20HE-enriched fraction was able to reduce stress, anxiety, and depression, in addition to maintaining antioxidant defenses of the cortex striatum.	[50]
The effects of 20HE in ovariectomized rats on morphological changes in the joint, epiphyseal cartilage, and trabecular tissue.	Female Wistar rats, n = 6/group: control, ecdysterone (3 g/kg food), and estradiol (10 mg/kg) groups.	Ecdysterone induced a significant increase in the thickness of joint cartilage and increased the whole epiphyseal growth plate and its proliferative and hypertrophic zones.	[51]
*β*-ecdysterone effects on osteoblast differentiation and bone regeneration in vitro and in vivo.	MC3T3-E1 cells. Fifteen male Sprague–Dawley rats. Rats with bone abnormalities (n = 10) were divided into two groups: intraperitoneal injections of PBS (n = 5) or 72 mg/kg of 20HE (n = 5), every three days.	*β*-ecdysterone is identified as a regulator for bone regeneration, can stimulate the BMP-2/Smad/Runx2/Osterix pathway and MC3T3-E1 cell regeneration and initiate bone rebuilding.	[74]
The effect of ecdysterone on lipid metabolism.	Male Wistar rats. Ecdysterone was injected intraperitoneally in a dose of 0.5 mg/kg, injection volume was 0.2 mL.	Ecdysterone: reduces free fatty acids, diglycerides, triglyceride lipase activity, and specific activity of phosphatidylcholine, increases the specific activity of phosphatidylethanolamine and phosphatidylserine in the liver.	[75]
Evaluate the effect of high-intensity interval training exercise (HIT) and ecdysterone consumption synergistically after Alzheimer’s disease.	72 adult male Wistar rats, 9 groups (n = 8 per group): control group, 10 mg/kg/day 20HE, performed HIIT or injection of Aβ peptide + 0.9% saline.	Due to the free radical scavenging and neuroprotective qualities, the combination of HIT exercise and ecdysterone therapy may be a viable therapeutic strategy for Alzheimer’s disease.	[76]
Extract from *A. turkestanica* or 20HE to sedentary aging mice would activate the key control point of protein synthesis.	Aging male C57BL/6 mice, 20 months old (n = 36). Vehicle group (n = 12), *A. turkestanica* extract (50 mg/kg/day; n = 12), or 20HE (50 mg/kg/day; n = 12). Duration: 28 days.	Treatment did not alter body, muscle, or organ mass; fiber cross-sectional area; or fiber type in the triceps brachii or plantaris muscles and muscle mass, nor did it activate protein synthesis.	[77]
20-hydroxyecdysone effects on obesity and diabetes.	H4IIE rat hepatoma cells. Six-week-old male C57BL/6J mice (n = 10/group): low-fat diet or a high-fat diet (HFD). The HFD animals randomized into: a control group (n = 10), and a treatment group (n = 10)—10 mg/kg/body 20HE. Duration: 13 weeks.	Anti-obesity and anti-diabetic effects of 20-HE.	[78]
Treatment with 20HE may reduce glucocorticoid-induced osteoporosis.	Male Swiss–Webster mice. Two groups (n = 8–10/group): placebo and prednisolone (3.3 mg/kg/day). Then separated into control group and 20HE (0.5 mg/kg/day). Duration: 21 days.	20-hydroxyecdysone showed increases in bone production and higher cortical bone mass. Concurrent treatment with 20HE and glucorticosteroids reduced bone formation rate, trabecular bone volume, and partially reversed cortical bone loss caused by glucocorticoid.	[79]
Examine ecdysterones derived from *R. carthamoides* neuroprotective mechanism of action.	In silico and 35 pathogen-free male Sprague–Dawley rats, aged 6–8 weeks. Seven groups from which 3 groups treated with ecdysterone (5, 10, and 20 mg/kg, respectively).	Neuroprotective effect and Removed glutamatergic excitotoxicity.	[80]
Ecdysterone effects on the cyclic AMP kinase system in mouse adipose tissue.	Male mice were injected intraperitoneally with 10 µg of ecdysterone or control mice received saline.	Decrease AMP-binding protein activity and cyclic AMP-dependent protein kinase.	[81]
Efficacy of 20-HE in ameliorating memory deficits within an animal model of type 1 diabetes mellitus.	Adult male Sprague–Dawley rats, divided into 2 control groups (n = 10) and animals (n = 70) in high-fat group were treated with a high-fat diet for 15 weeks. Other groups were received 20HE (1, 10, and 100 mg/kg/day) for 12 weeks.	20-hydroxyecdysterone has a protective role in memory deficits in rats with diabetes mellitus.	[82]
Effect of 20HE on the osteogenic differentiation ability of bone marrow mesenchymal stem cells.	Human bone marrow mesenchymal stem cells and 40 Sprague–Dawley rats (5 groups): control group, model group, 20HE: 40 mg/kg, 20 mg/kg) and 10 mg/kg for 14 days.	*β*-ecdysterone may be beneficial for the recovery of osteonecrosis of bone marrow mesenchymal stem cells.	[83]
Effects of ecdysterone on cAMP and cGMP levels in mouse plasma.	Male mice, ecdysterone was given intraperitoneally at a dosage of 10 μg/animal.	The heterophilic effects of ecdysterone in mammals may be facilitated by modulation of the cyclic AMP system.	[84]
Effects of ecdysterone on cAMP and cGMP levels in mouse liver.	Male mice, ecdysterone was given intraperitoneally at a dosage of 10 μg/animal.	The cyclic AMP-protein kinase system is likely implicated in mediating the heterophilic effects of ecdysterone.	[85]
Investigation the underlying molecular mechanisms, in particular the role of estrogen receptor beta.	Male Wistar rats, groups (n = 6) treated for 21 days with 5 mg/kg 20HE or vehicle.	Ecdysterone led to an increase in muscle fiber size, accompanied by elevated serum insulin-like growth factor 1 (IGF-1) levels and decreased levels of corticosterone and 17β-estradiol (E2). Hypertrophy was induced by treatment with 20HE, dihydrotestosterone, IGF-1, and E2.	[86]
Ecdysterone lipid-lowering effects in obese Zucker rats.	16 male, homozygous obese Zucker rats and 16 male, 25-week-old, heterozygous Zucker rats. Two groups (n = 8/group). The obese rats were allocated to two groups (n = 8/group): control and ecdysterone (0.5 g/kg/food).	Ecdysterone supplementation lacks lipid-lowering effects in liver and plasma of lean and obese Zucker rats.	[87]
Effects of 20HE on CYP11B1/2 levels and activity in UVB-induced photoaging and skin lesions in hairless mice.	PC12 cell line, HaCaT cell line, and male hairless mice (CrlOri:SKH1), 20HE or osilodrostat administered orally for 7 days.	20-hydroxyecdysone prevents UVB-induced skin aging by inhibiting aldosterone synthase. It is a promising candidate for anti-aging therapy.	[88]
Effect of *β*-ecdysterone on osteogenic differentiation of bone marrow mesenchymal stem cells and its dependents on the estrogen receptor.	MSCs isolated from 8- to 10-week-old male Babl/c mice. Control, all-trans-retinoic acid, and ecdysterone groups. β-ecdysterone (1 mg/kg body weight) was administered intravenously daily for 3 weeks.	In vitro, β-ecdysterone stimulated osteogenic differentiation of mesenchymal stem cells in an estrogen receptor-dependent way, reducing osteoporotic processes in a mouse model.	[89]
20-hydroxyecdysone for improving skin conditions in postmenopausal women, investigation in ovariectomized rats.	After ovariectomy rats. 20HE (18, 57, or 116 mg/animal/day) or 17A-estradiol-3-benzoate (60 Kg/kg body weight) for 12 weeks.	Ecdysterone prevented the ovariectomy-induced decrease in subcutaneous musculature, contrasting with the effects of 17*β*-estradiol.	[90]
Effect of 20HE on oxidative stress and inflammation in a collagen-induced rheumatoid arthritis rat model.	Forty healthy male Sprague–Dawley rats, (4 gropus, n = 10). Rats given saline for 28 days. Groups I and II: collagen-induced arthritis-induced rats and groups III and IV: rats treated with 10 or 20 mg/kg body weight 20HE for 28 days.	Administering 20HE (20 mg/kg) to collagen-induced rheumatoid arthritis rat models successfully reduces inflammation and oxidative stress, resulting in anti-rheumatoid arthritis effects.	[91]
Ability of 20HE to activate mTORC1 signaling in both skeletal muscle and liver tissues.	Male Sprague–Dawley rats. Dose-response study: Rats were separated into groups (n = 5–6) and administered doses of 0, 10, 50, or 200 mg/kg of 20HE via gavage. Time-course study: Rats were separated into groups and given either 200 mg/kg of 20HE or an excipient. Combination study: Rats were separated into groups and given either an excipient, 200 mg/kg of 20HE, or 200 mg/kg of 20HE plus 1.35 g/kg L-leucine by gavage.	20-hydroxyecdysone does not quickly activate mTORC1 signaling in muscle or liver.	[92]
Ecdysterone as a modulator of cytostatic therapy.	Hybrid mice BDF1. On days 2, 4, 6, and 8, mice were given cisplatin (2 or 4 mg/kg) or cisplatin with ecdysterone (2 and 10 mg/kg).	Ecdysterone enhanced the chemotherapeutic impact of low doses of cytostatic.	[93]
Ethanol extract from *Pfaffia glomerata* and isolated ecdysterone hypnotic effect.	Adult male Wistar rats and adult male CF1 mice. Treatments: saline i.p. (n = 18); first—saline + polysorbate 80 1% intra peritoneal (n = 13); aquas 500 mg/kg i.p. (n = 9); butanol 500 mg/kg i.p. (n = 9); oranic 1 -500 mg/kg i.p. (n = 10); diazepam 1 mg/kg i.p. (n = 16).	The lipophilic fraction derived from *P. glomerata* demonstrates a hypnotic effect, with ecdysterone identified as one of the compounds responsible for this central nervous system activity.	[94]
“Ecdysterone-80”—an extract of phytoecdysteroids from *Serratula coronata* L., dominated by 20HE (81–86%).	168 male rats. Ecdysterone-80 at a dosage of 20 mg/kg for 60 days beginning in the second month of the trial.	Ecdysteroids confer a cardioprotective effect.	[95]
*Stachys hissarica* and isolated ecdysteroids (ecdysterone etc.) effect on skin wounds healing.	Rats were orally administered the extract at a repeated dose of 10 mg/kg.	The ecdysteroid-containing preparation derived from *S. hissarica* exhibited notable effectiveness in promoting wound healing activity.	[96]
Ecdysterone derived from *Achyranthes bidentate* and Paeonol derived from *Cortex Moutan.*	Male Sprague–Dawley rats. 10% ecdysterone and 5% paeonol. Rats in each group were given vehicle or compound treatments three times a day. Duration: 12 days.	Orally given ecdysterone-paeonol reduced radiation-induced oral mucositis in rats.	[97]
20-hydroxyecdysone isolated from *Silene viridiflora* influence on size of the different muscle fiber types	Twenty male Wistar rats (5 groups, n = 4/group): 0.9% NaCl, a daily subcutaneous injection of 5 mg/kg body weight of 20HE, snake venom (notexin) injections. From the 5th day post-injection: a daily subcutaneous injection of 5 mg/kg body mass weight of 20HE for 7 days or a daily subcutaneous injection of 0.5 mg/kg body mass weight of 20HE for 7 days.	20-hydroxyecdysone also augmented the myonuclear count within the fibres of both normal and regenerating muscles.	[98]
20-hydroxyecdysone from the dietary supplement “Peak Ecdysone”.	Fourty-six men (mean age 25.6 ± 3.7 years). Twenty men took 200 mg of 20HE, ten took 800 mg, and twelve received a placebo. Control group—12 volunteers consumed 200 mg of 20HE without training. Duration: 10 weeks.	20-hydroxyecdysone increases body weight and muscle mass. Increased power and strength in performance. No adverse impacts on creatinine, gamma-glutamyl transferase, glutamate-oxaloacetate transaminase, or glutamate-pyruvate transaminase. The steroid profile was not affected.	[26]
Ecdysterone, a spinach component, has the potential to prevent metabolic syndrome.	Overweight women and men (18 receiving placebo, 21 receiving verum) consumed 2 × 50 mg of 20HE, along with 2 × 450 mg of spinach powder, daily.	It has been determined that this product can be utilized to treat and prevent the development of metabolic syndrome.	[99]
20-hydroxyecdysone—randomised double-blind Phase 1 study for safety, tolerance pharmacokinetics and pharmacodynamics in healthy young and older adults (age ≥ 65 years).	Part 1: Single ascending dose (SAD): Three cohorts: A, B, and C. Cohorts A and B each had eight young individuals ranging in age from 18 to 55. Cohort C consists of eight senior participants (65 years or older). In the fasting state, subjects were given escalating dosages of BIO101 or a placebo. Part 2: Multiple ascending doses (MAD): Dose levels based on Part 1 findings. Three cohorts of ten older people (aged 65 to 85). Subjects were randomized to receive BIO101 or a placebo once or twice daily.	BIO101 (20HE) is well tolerated at doses of 350 mg/day, 350 mg twice a day, and 450 mg twice a day. Showed good safety profile in young and older adults, without serious adverse effects.	[100]
Methoxyisoflavone, 20HE and sulfopolysaccharides Intake results on training adaptation.	45 resistance-trained males: group 1—placebo, group 2—800 mg/day of methoxyisoflavone, group 4—200 mg of 20HE, and group 5—1000 mg/day of sulfopolysaccharide. Duration 8 weeks.	No changes were observed in training adaptation and in anabolic/catabolic effect.	[101]

**Table 3:** *Rhaponticum carthamoides* in vitro studies.

Study Objectives	Cell Lines	Main Results	Ref.
Anti-adipogenic activity of *R. carthamoides* extract, ecdysterone, turkesterone and ponasterone A.	SGBS human adipocytes.	*Rhaponticum carthamoides* extract, ecdysterone and turkesterone reduced lipid accumulation in human adipocytes and *R. carthamoides* and ecdysterone stimulated lipolysis.	[69]
Influence of *R. carthamoides* transformed roots extract on the growth of grade II and III human glioma cells.	Human primary glioma cell lines (grade II and III astrocytoma).	The extract from *R. carthamoides* transformed roots shows anticancer properties by impeding the proliferation of glioma cells and prompting apoptotic cell demise.	[121]
The cytotoxic effect and apoptotic potential of extracts derived from *R. carthamoides* transformed roots and roots of soil-grown plants evaluated in human glioma primary cells.	Human glioma cancer cells and normal human astrocytes.	*Rhaponticum carthamoides* root extracts reduce cell proliferation and promote apoptosis in human glioma cells.	[122]
Efficacy of *R. carthamoides* against myocardial injury.	A myocardial ischaemia in male SD rats and H9c2 cell lines.	*Rhaponticum carthamoides* possess cardioprotective effect.	[123]
Antimicrobial activity of *R. carthamoides* and *Potentilla fruticosa* L. extracts.	*Escherihia coli*, *K. pneumoniae*, *P. aeruginosa*, *Proteus mirabilis*, *Enterococcus faecalis*, *S. aureus*, *Bacillus subtilis*, *B. cereu*, and *C. albicans*	*Rhaponticum carthamoides* and *Potentilla fruticosa* extracts, exhibit antimicrobial properties against *E. coli*, *K. pneumoniae*, *P. aeruginosa*, *E. faecalis*, *B. subtilis*, *B. cereus*, *S. aureus*, and *C. albicans*.	[124]
20-hydroxyecdysone, *R. carthamoides* extracts, and selected steroidal/non-steroidal anti-inflammatory drugs influence on NF-κB-inhibiting activity.	Human cervical cancer HeLa-IL-6 cells.	The extracts showed a significant modulation of the NF-κB inhibitory effect of dexamethasone.	[125]
*Rhaponticum carthamoides* root extract and 20HE, effects on human breast adenocarcinoma.	Human breast adenocarcinoma MCF-7 cells.	20-hydroxyecdysone does not affect cell proliferation, ERα protein, or 5α-reductase activity. The extract inhibited cell proliferation and affected AhR-agonistic activity.	[126]
Extracts from *R. carthamoides* transform roots and soil-grown plant roots’ effect on oxidative stress.	Chinese hamster ovary (CHO) cells.	*Rhaponicum carthamoides* possess antioxidant properties protecting CHO cells from oxidative stress.	[127]
Compare the composition and antioxidant activity of *R. carthamoides* leaf extracts taken from plants growing in Poland and Russia.	HL-60 cells (human leukaemia).	The extracts from the Polish material had better antioxidative and cytotoxic activity than those from the Russian.	[128]
The effect of *R. carthamoides* on the proliferation of a diverse population of rumen bacteria and their influence on the fermentation process of a feed mixture in an artificial rumen (Rusitec).	Rumen bacteria and artificial rumen (RUSITEC)	No significant influence of *R. carthamoides* on rumen microflora metabolism, nor any effect of stimulative chemicals found in the plant’s above-ground parts.	[129]
The antibacterial activity of crude ethanol extracts from the aerial parts and roots of four leuzea species incl. *Leuzea carthamoides* DC.	*Bacillus subtilis*, *B. cereus*, *Bacteroides fragilis*, *E. faecalis*, *E. coli*, *P. aeruginosa*, *S. aureus*, *Staphylococcus epidermidis*, *Streptococcus pneumoniae*, and *Streptococcus pyogenes.*	Extracts from aerial parts of all examined species showed notable antibacterial activity, particularly against *B. cereus*, *S. epidermidis*, and *B. fragilis*. *L. carthamoides* was the most efficient.	[130]
Antimicrobial activity of crude ethanolic extracts derived from 16 Siberian medicinal plants (incl. *R. carthamoides*)	*Escherihia coli*, *B. cereus*, *P. aeruginosa*, and *Salmonella enteritidis*, *C. albicans*	*Rhaponticumcarthamoides* affect mostly *Bergenia crassifolia* and *Staphylococcus aureus* at MIC 15.63 and 62.50, respectively.	[131]
The effects of four adaptogenic plants (inkl. *R. carthamoides*) extracts on the life span.	*Philodina acuticornis* bdelloid rotifers.	Ajugasterone C from *R. carthamoides* exhibited lower toxicity than 20HE.	[132]

**Table 4:** *Rhaponticum carthamoides* in vivo studies and trials.

Study Objectives	Study Design	Main Results	Ref.
20-hydroxyecdysone isolated from *Achyranthes faurei* influence on glucose levels.	Male Donryu rats and male ddy mice, alloxane-induced. Ecdysterone injected 0.1, 0.5, 1 and 10 mg/kg.	Ecdysterone did not affect blood glucose levels in non-diabetic animals but reduced blood glucose levels in alloxan-diabetic mice.	[44]
*Rhaponticum carthamoides* extract effects on rats’ learning and memory processes.	Rats were orally administered *R. carthamoides* extract at doses of 0.25, 0.5, and 2.5 g/kg body weight for 10 days preceding the training sessions.	The extract increased learning and memory indices to varied degrees, depending on the amount used.	[133]
The central neurotropic activity of an aqueous-alcoholic extract from *R. carthamoides* cultivated in Bulgaria.	Male Wistar rats. The extract in doses of 200, 500, and 1000 mg/kg was injected subcutaneously 1 h prior to or immediately after training. Effect of extract on blood pressure conducted on male cats weighing 3.0 to 4.0 kg, administered intraduodenally in 1 mL/kg body weight.	The extract of *R. carthamoides* showed significant central stimulating effects, including enhanced ambulation increased central nervous system excitability, and a minor antinarcotic action and rearing behavior, improved learning, memory ability, and physical endurance in rats.	[134]
*Rhaponticum carthamoides* extract effects compared to those of *Glycyrrhiza glabra* and *Punica granatum extracts.*	Male Wistar Albino Glaxo rats incorporated with 300 mg/kg/day extracts.	*Rhaponticum carthamoides* extract powder supplementation significantly enhanced glucose and lipid metabolism compared to *Glycyrrhiza glabra* and *Punica granatum* extracts.	[135]
Investigate the impact of *R. carthamoides*, *R. rosea*, and their combined administration on resistance exercise and mechanical power.	Eleven-week-old Wistar Han rats (n = 56), 7 groups (n = 8). Rhodiola group—43.5 mg/kg body weight; *R. carthamoides* group—43.5 mg/kg body weight; *R. carthamoides* and Rhodiola groups 1–4 87.1, 43.5, 21.8. 8.7 43.5 mg/kg body weight, respectively.	*R. carthamoides* extract promotes muscle protein synthesis. The combination with *R. rosea* improved muscle protein synthesis and power performance.	[136]
Ecdysterone from the roots of *R. carthamoides,* influence on the mitochondrial level.	White male mongrel rats. Ecdysterone was administered orally to experimental group for 10 days before the start of the experiment at a daily dose of 5 mg/kg.	Ecdysterone may directly influence mitochondrial bioenergetics.	[137]
Ecdysterone isolated from *Rhaponticum integrifolium*, turkesterone isolated from *A. turkestanica.*	Male mongrel white mice subjected to immobilization on their back for 6 h. Ecdysterone, turkesterone, and T-activin were administered intraperitoneally before immobilization.	Ecdysterone and turkesterone prevented and facilitated the consequences of stress and restored the immunological activity.	[138]
Genotoxic evaluation of ***β***-ecdysone.	Twelve-week-old male Wistar rats. 0.0823, 0.4115, and 0.8230 mg/kg treatments.	The results suggest cytogenotoxic activity of 2HE.	[139]
Effect of *R. carthamoides* extract on the lipid content of the erythrocyte membrane in rats with cerebral ischemia.	Male Wistar rats (n = 18). *R. carthamoides* extract administration (150 mg/kg).	*Rhaponticum carthamoides* extract increased the total lipid and phospholipid contents.	[140]
The impact of different levels of *R. carthamoides* hay meal in rat diets.	30 female and 30 male SPF Wistar rats, 10 groups (n = 6). Hay meal with *R. carthamoides* 5–50%.	The use of a diet containing 20% *R. carthamoides* has resulted in significant variability in teste weight.	[141]
Effect of an herbal supplement comprising a 70:30 combination of extracts *R. carthamoides* and *R. rosea* on performance fatigue.	Thirty healthy active men (age 22.3 ± 4.1 years): 350 mg dose of the DS, a 175 mg dose of the DS with 175 mg of maltodextrin, or a placebo—350 mg (maltodextrin).	No significant influence on performance fatigability during high-intensity, repetitive muscular activities.	[142]

**Table 5:** *Spinacia oleracea* in vivo studies.

Study Objectives	Study Design	Main Results	Ref.
Mechanism of action of PEs (20-HE, polypodine B, and ponesterone) and *A. turkestanica* and *S. oleracea* in mammalian tissue, focusing on protein synthesis and physical performance.	A skeletal muscle cell line (mouse), C2C12 (ATCC CRL-1772)—threatened with 20HE, turkesterone, ponesterone, polypodine B, methandrostenolone, *A. turkestanica* and *S. oleracea* extract. Male Sprague–Dawley rats, divided in four groups: vehicle, 50 mg/kg 20HE, 1000 mg/kg spinach extract, or 10 mg/kg methandrostenolone given orally. Duration: 28 days.	In vitro, PEs increased protein synthesis in C2C12 murine and human primary myotubes. Ecdysteroids improved grip strength in rats, according to in vivo research. Similarly, plant extracts containing ecdysteroids had comparable results.	[19]
Anti-hyperlipidaemic and anti-obesity effects of *S. oleracea* extract.	Sprague–Dawley female rats (7 groups, n = 6): control—normal diet, high fed diet control (HFD), orlistat (10 mg/kg), antioxidant-rich extract of *S. oleracea* (200 mg/kg, 400 mg/kg, p. o), group 4—HFD and aerobic exercise (AE) for 20 min daily, group VII (NAOEAE)—NAOE (400 mg/kg, p. o) 20 min before receiving HFD and AE for 20 min daily. Duration: 21 days.	*S. oleracea* extract demonstrated significantly reduced hyperlipidaemia. The combined therapy and aerobic exercise, underscored the importance of incorporating both exercise and antioxidant-rich dietary sources to managed lipid levels and obesity.	[164]
A flavonoid-rich extract from *S. oleracea* leaves effect on appetite in rats.	Sprague–Dawley female rats, 4 groups (n = 6) Control—received drinking water (1 mL/kg, per oral, reference standard—received fluoxetine (6 mg/kg, intraperitoneal), *S. oleracea* extract 200 mg/kg, or 400 mg/kg per oral. Duration: 14 days.	Spinach leaves extract shows promising appetite suppression effect.	[165]
Protective effect of spinach against radiation-induced oxidative stress.	Male Swiss albino mice, four groups (n = 30). Normal group, fed with spinacia extract (1100-mg/kg body weight/day or 1100-mg/kg body weight/day), and control group. Duration: 15 days.	*Spinacia oleracea* showed strong protective effects against radiation-induced oxidative stress in mouse livers, indicating its potential as a low-cost source of antioxidants.	[166]
Effect of *Spinacia oleracea* extracts on ulcer regeneration, particularly in diabetic conditions.	Seventy-two male Sprague–Dawley rats, six groups (n = 12). Group 1—diabetic rats—300 mg/kg saline. Group 2—non-diabetic rats—300 mg/kg normal saline. Group 3—diabetic rats—300 mg/kg *S. oleracea* aquatic extract (SOAE). Group 4—diabetic rats—300 mg/kg SOAE. Group 5—intact rats—300 mg/kg SOAE. Then, they were exposed to diabetes and received 300 mg/kg SOAE. Group 6—intact rats—300 mg/kg *S. oleracea* alcoholic extract. Then, they were exposed to diabetes and received 300 mg/kg *S. oleracea* alcoholic extract. Duration: 1 month.	*Spinacia oleracea* extracts may be effective in the regeneration of diabetic ulcers.	[167]
Anti-asthmatic activity of *S. oleracea* an aqueous extract.	B16 cells and SH-SY5Y neuroblastoma cells and ovalbumin-induced asthmatic mouse model: Mice weighing 25 ± 2.5 g, male, 7-week, the aqueous *S. oleaceae* extract.	In the ovalbumin-induced asthmatic mouse model, the aqueous spinach extract efficiently alleviates asthmatic symptoms.	[168]
Spinach—antioxidant and hyperlipidaemic effect.	HepG2 cells and male Sprague–Dawley rats (n = 24). Three groups of eight animals each. Normal diet, a high fat-cholesterol diet (HFCD), or a HFCD supplemented with 5% freeze-dried spinach powder.	Spinach led to improvements in lipid profiles, decreased oxidative stress markers in the liver and blood, and enhanced antioxidant enzyme activity.	[169]
Examined the preventive efficacy of spinach natural antioxidants.	B16 melanoma cell line and female Balb/c mice. Saline—treated controls; doxorubicin (DOX) injected intraperitoneally -20 mg/kg, group 2—spinach natural antioxidant (NAO) administration i.p. for 7 days before and for 6 days after DOX administration (130 mg/kg); group 3—NAO administration i.p. for 6 days after DOX administration (60 mg/kg); and NAO administered i.p. for 13 days (cumulative dose, 130 mg/kg).	Water-soluble antioxidants from spinach can reduce doxorubicin-induced cardiotoxicity without adverse effects.	[170]

**Table 6:** *Spinacia oleracea* randomized controlled trials.

Study Objectives	Study Design	Main Results	Ref.
The influence of chronic daily spinach supplementation on oxidative stress and muscle damage.	Twenty well-trained men, randomised in two groups: spinach (n = 10) and placebo (n = 10). The participants took spinach supplementation (1 g/kg body weight) or placebo daily for 14 days before running (21.1 km).	Persistent daily oral supplementation with spinach reduces indicators of oxidative stress and muscle injury after a half-marathon in well-trained healthy young men.	[162]
Effects of daily supplementation with a *S. oleracea* extract with a moderate-intensity exercise programmed on skeletal muscle.	Over 50-year-old adults (Caucasian men and postmenopausal women aged between 50 and 75 years). Daily intake of a spinach extract for 12 weeks and a 12-week physical exercise resistance training program on skeletal muscle.	Training combined with daily intake of *S. oleracea* extract may increase muscle-related factors and muscle quality.	[171]
Benefits of spinach, in a therapeutic diet for obese and insulin-resistant patients.	Fourteen normal-weight and ten obese men, 20 to 35 years, consumed three test meals of bread, as a control, bread and butter, and bread and butter with boiled spinach.	Green leafy vegetable intake with a fat-rich meal may effectively supply postprandial α-tocopherol in obese subjects.	[172]
Supplementation with thylakoid membranes from spinach, combined with a hypocaloric diet, effect on lipopolysaccharide (LPS) levels, neurotrophic factors, and oxidative stress in polycystic ovary syndrome (PCOS).	Forty-eight obese women diagnosed with PCOS, aged 20–45 years: thylakoid (n = 21) and placebo groups (n = 23). Supplementation with a thylakoid-rich spinach extract (5 g/day) or a placebo (5 g cornstarch. Duration: 12 weeks.	Supplementation with thylakoid from spinach, combined with a hypocaloric diet, resulted in reduced LPS levels, increased neurotrophic factor levels, and improved glycaemic and sex hormone profiles in PCOS patients.	[173]
High-intensity functional training (HIFT) combined with spinach-derived thylakoid supplementation effect on selected adipokines and insulin resistance in males with obesity.	Sixty-eight participants, mean age: 27.6 ± 8.4 years, four groups (n = 17): control group, supplement group, training group, and training with supplement group. The two training groups started the 12 weeks of exercise training program. The eligible participants received 5 g/day of thylakoid-rich spinach extract or matching placebo as 5 g/day of raw corn starch. Duration: 12 weeks.	Potential benefits of combining HIFT with spinach-derived thylakoid supplementation in improving adipokine profiles and insulin resistance in males with obesity over a 12-week period.	[174]
The impact of taking a single dosage of concentrated extract of thylakoids from spinach on satiety, food intake, lipids, and glucose.	Sixty overweight and obese individuals aged 18–65 years. They consumed the spinach extract or placebo. Blood was drawn for assessments of lipids and glucose before a breakfast meal, followed 4 h later by a 5 g dose of the extract and a standard lunch. Two hours after lunch a second blood draw was conducted.	Consuming the spinach extract significantly reduced hunger. Postprandial plasma glucose concentrations were increased following. There were no differences in plasma lipids and energy intake at dinner.	[175]
Carotenoid-rich leafy vegetables—spinach and perilla effect on carotenoid concentration and oxidative stress in human blood plasma.	Twelve volunteers, aged 19–44 years. Examination after a 10-day consumption of perilla or spinach, after the washout phase (10 days), and after the following 10-day consumption. Duration: 10 days.	Significant increases in lutein content were observed in human blood plasma after consumption of both spinach and perilla preparations. Both perilla and spinach preparations influence lipid peroxidation.	[176]
Calcium possibility to affect bioavailability of carotenoids from a vegetable matrix—spinach.	Twenty-five non-obese men aged 20–46 years. Each participant received 270 g of spinach-based meals (8.61 mg carotenoids/100 g fresh weight), 0, 500, or 1000 mg of Ca.	Calcium did not affect the bioavailability of carotenoids from a spinach-based meal.	[177]
To characterize tissue lutein and β-carotene concentrations in various spinach cultigens, and to determine serum carotenoid and macular pigment optical density (MPOD) responses in human subjects consuming spinach cultigens.	Thirty individuals (21–60 years). Three groups (n = 10/group): control group, high-lutein spinach group, and low-lutein spinach group.	Consumption of low-lutein spinach led to a 22% increase in average serum lutein concentrations, while consumption of high-lutein spinach resulted in a 33% increase, and consuming high-lutein spinach demonstrated significant increases in MPOD.	[178]

**Table 7:** *Chenopodium quinoa* in vitro studies.

Study Objectives	Cell Lines	Main Results	Ref.
Quinoa and its polysaccharides effects on the gut microbiota.	In vitro, including three different stages: simulated oral, gastric, and small intestine digestion.	Quinoa and its polysaccharides improved microbiota composition and short-chain fatty acid synthesis. They are potential prebiotics.	[201]
The effect of *C. quinoa* leaves phenolic compounds on cancer cells.	Rat prostate cancer AT-2 and MAT-LyLu, HTB-140 and normal mouse 3T3 fibroblasts.	The phenolic compounds may exert a chemopreventive and anticarcinogenic effect on oxidative stress.	[202]
The polyphenol composition and antioxidant properties of methanolic extracts from amaranth, quinoa, buckwheat, and wheat.	Antioxidant capacity is determined by using the radical DPPH scavenging capacity assay.	Total phenol content and antioxidant activity were shown to rise after sprouting, but levels decreased after breadmaking.	[203]
The presence and bioactivity of lunasin in quinoa.	RAW 264.7 macrophage cells	The lunasin isolated and purified from quinoa inhibited the production of nitric oxide, tumor necrosis factor-α, and interleukin-6.	[204]
Antioxidant, anti-diabetic, and immunoregulatory activities of the isolated polysaccharides from quinoa seeds.	Antioxidant activities (DPPH radical scavenging activity and ABTS radical scavenging activity. Antidiabetic activities (alpha-glucosidase inhibitory activity and alpha-amylase inhibitory activity), RAW264.7 cells for immunostimulatory activities.	Dose-dependent antioxidant and antidiabetic activities of the polysaccharides isolated from quinoa seeds, along with immunoregulatory activity.	[205]
The inhibition of collagenase activity of quinoa extracted ecdysteroids.	DPPH free-radical-scavenging. Collagenase inhibition assay—method of Sawabe.	The potential of ecdysteroids from quinoa in modulating collagenase activity.	[206]
In vitro antiplatelet activity of quinoa and lupin bean extracts.	Six healthy young male volunteers (ages 20–30) participated in the study. 10 mL venous blood samples were obtained for platelets.	Quinoa extracts did not exert an anti-aggregatory effect, whereas lupin extracts showed a significant effect.	[207]

**Table 8:** *Chenopodium quinoa* in vivo studies.

Study Objectives	Study Design	Main Results	Ref.
Effects of hydrolyzed quinoa.	Wistar rats (n = 64): control groups, treated group with hydrolyzed quinoa 2.000 mg/kg and treated and exercised group for 30 days.	Hydrolyzed quinoa decreased body weight, blood triacylglycerol level, and fat deposition.	[200]
The ability of quinoa extract enriched in 20-HE to prevent obesity in mice.	Male C57BL/6J mice (6-week-old), 4 groups (n = 12/group). The high-fat group was supplemented or not with either quinoa extract or 20HE (6 mg/day/kg body weight).	Quinoa possesses anti-obesity activity; similar effect was observed with ecdysterone.	[208]
Anxiolytic and antioxidant effects of PEs (incl. 20-HE) and polyphenols from *C. quinoa.*	Sixty male Wistar rats. Three groups (n = 12): CTRL, IMM, and IMM-FFI. The CTRL and IMM groups were treated with a standard half-synthetic diet for 36 days. The third group, IMM-FFI, was treated with a standard diet with the addition of FFIs in the dose of 0.055 ± 0.003%.	The nutritional support from *C. quinoa* effectively normalized malondialdehyde and catecholamine levels under induced oxidative/nitrosative stress, while maintaining high superoxide dismutase activity, improving behavioral reactions and cognitive functions.	[209]
Hypoglycemic activity of quinoa leachate (QL).	Five-week-old male C57Bl/6J mice. Mice were randomly divided into an oral ingestion of 0.25 mL/50 g body weight of vehicle (70% Labrasol^®^) or QL (250 and 500 mg/kg) (n = 7). Metformin (300 mg/kg) was administered as a positive control.	The optimized quinoa leachate, including 0.86% 20-HE, 1.00% total Pes, 2.59% flavonoid glycosides, 11.9% oil, and 20.4% protein, notably reduced fasting blood glucose levels in obese, hyperglycaemic mice.	[210]
The impact of chronic intake of quinoa extract and 20HE on the regulation of energy homeostasis in mice.	Male C57BL/6J mice (6-week-old). Fed with a high-fat (HF) diet, supplemented or not with quinoa extract (0.28%) or 20HE (6 mg/day/kg body weight) for 3 weeks.	Both quinoa extract and 20HE promoted a higher rate of glucose oxidation. Quinoa extract possesses an anti-obesity effect.	[211]
Investigate the potential weight management effects of hydroalcoholic extracts from quinoa seeds and chia (*Salvia hispanica* L., Lamiaceae), and assess the hepatoprotective, anti-inflammatory, and antioxidant activities.	Albino Wistar rats, five groups (n = 6/group): normal food diet (NFD)—normal food diet and saline, high-fat diet (HFD)—high-fat diet and saline, (HFD and green tea)—HFD and green tea extract (250 mg/kg/day), (HFD and quinoa)—HFD and quinoa (250 mg/kg/day), (HFD and chia)—HFD and chia (250 mg/kg/day) for 6 weeks.	Chia and quinoa seed extracts showed hepatoprotective, anti-inflammatory, and antioxidant activity in obese rats given a high-fat diet. These extracts also influenced important metabolic indicators such as leptin, adiponectin, serum lipids, and glycaemic levels.	[212]
Effect of quinoa on intestinal permeability and inflammation.	BN rats and Caco-2/TC7 cells. BN rats with an age of 7–12 weeks, five groups (n = 24). Daily administered for one week: control group, quinoa flour (300 mg) with or without control, or capsaicin (12 mg).	Quinoa has the ability to enhance intestinal permeability and promote compartment-specific protein uptake, potentially through mechanisms distinct from those of cholera toxin and capsaicin.	[213]
Polyphenol composition of red and yellow quinoa seeds, effect of antioxidant bioactivity of both raw and germinated quinoa seeds in vitro and effect against carbon tetrachloride (CCl4)-induced oxidative stress in rats.	Markers of oxidative stress were evaluated in Wistar rats, which were categorized into five groups, each comprising six rats. Group I received an intraperitoneal injection of fresh olive oil (1.0 mL/kg/twice per week) along with 0.5 mL of distilled water orally per day. Group II received an intraperitoneal injection of a fresh mixture of CCl4 and olive oil (1.0 mL/kg/twice per week). Group III was administered an intramuscular injection of vitamin E and selenium (at a dosage of 30 mg/kg/twice per week) in combination with CCl4. Group IV was given an oral dose of 30 mg/kg gallic acid-yellow quinoa extract for 4 weeks concurrently with CCl4. Lastly, group V received 30 mg/kg gallic acid-red quinoa extract for 4 weeks along with CCl4.	Both red quinoa sprouts and yellow quinoa sprouts contain naturally synthesized polyphenols with superior antioxidant activity. The ethanolic extracts of quinoa sprouts demonstrated promising effects in counteracting oxidative stress.	[214]

**Table 9:** Turkesterone studies.

Study Objectives	Study Design	Main Results	Ref.
Antiadipogenic activity of *R. carthamoides* extract, ecdysterone, turkesterone, and ponasterone A.	SGBS human adipocytes.	*Rhaponticum carthamoides* extract, ecdysterone, and turkesterone reduced lipid accumulation in human adipocytes and *R. carthamoides-* and ecdysterone-stimulated lipolysis.	[69]
Ecdysterone isolated from *Rhaponticum integrifolium*, turkesterone isolated from *A. turkestanica.*	Male mongrel white mice subjected to immobilization on their back for 6 h. Ecdysterone, turkesterone, and T-activin were administered intraperitoneally.	Ecdysterone and turkesterone prevented and facilitated the consequences of stress and restored immunological activity.	[138]
Turkesterone isolated from the roots of *A. turkestanica* and its anabolic effect.	Sexually immature male rats weighing 60–80 g. Turkesterone was administered orally at a dose of 5 mg/kg. For comparison, nerobol (methandrostenolone) was used in a dose of 10 mg/kg.	Turkesterone exhibited pronounced anabolic, leading to significant increases in body weight, comparable in anabolic effect to nerobol. It did not exhibit androgenic effects.	[219]
The impact of turkesterone on the endocrine and exocrine function in alloxan-induced diabetic rats.	White outbred rats, divided into 4 groups: saline group and single dose of alloxan group (170 mg/kg, intraperitoneal) and/or turkesterone groups (10 mg/kg/24 h) and glibenclamide (5 mg/kg/24 h) for 10 days.	Turkesterone improved the exocrine and endocrine function in alloxan-induced diabetic rats.	[225]

**Table 10:** *Ajuga turkestanica* studies.

Study Objectives	Study Design	Main Results	Ref.
Mechanism of action of Pes (20HE, polypodine B, and ponesterone) and *A. turkestanica* and *S. oleracea* in mammalian tissue, focusing on protein synthesis and physical performance.	A mouse skeletal muscle cell line, ‘C2C1’ (ATCC CRL-1772)—threatened with 20HE, turkesterone, ponesterone, polypodine B, methandrostenolone, *A.turkestanica*, and *S. oleracea* extract. Male Sprague–Dawley rats, divided into four groups: vehicle, 50 mg/kg 20HE, 1000 mg/kg spinach extract, or 10 mg/kg methandrostenolone given orally. Duration: 28 days.	In vitro, phytoecdysteroids increased protein synthesis by up to 20% in C2C12 murine and human primary myotubes. Ecdysteroids improved grip strength in rats, according to in vivo research. Plant extracts containing ecdysteroids had comparable results.	[19]
*Ajuga turkestanica*, isolated compounds (including ecdysterone and turkesterone) antioxidant, cytotoxic, and antimicrobial activities.	HeLa, HepG-2, and MCF-7 human cell lines. Growth of *Enterococcus VanB* VRE ATCC 902291, *E. VanB* VRE ATCC 31299, *Staphylococcus aureus* MRSA ATCC 1000/93, and *S. aureus* MRSA ATCC 10442, *S. aureus* MRSA ATCC 10442, *S. pyogenes* ATCC 12344, *C. albicans* ATCC 90028, *Candida glabrata* ATCC MYA 2950, *K. pneumonia* ATCC 700603, and *P. aeruginosa* ATCC 27853.	The isolated ecdysteroids and iridoid glucosides from *A. turkestanica* were found to lack antioxidant properties and did not exhibit significant cytotoxic or antimicrobial effects. Interestingly, the chloroform extract, containing more lipophilic compounds, demonstrated higher activity.	[59]
Extract from *A. turkestanica* or 20HE to sedentary aging mice would activate the key control point of protein synthesis.	Aging male C57BL/6 mice, 20 months old (n = 36). Mice were randomly assigned to one of three treatment groups: vehicle (n = 12), *A. turkestanica* extract (50 mg/kg/day, n = 12), or 20HE (50 mg/kg/day, n = 12). Duration: 28 days.	Treatment did not alter body, muscle, or organ mass; fiber cross-sectional area; or fiber type in the triceps brachii or plantaris muscles. Phytoecdysteroid treatment does not alter muscle mass or fibre type.	[77]
Phytoecdysteroid enriched extract from *A. turkestanica* affects Notch and Wnt signaling in aged skeletal muscle.	Male C57BL/6 mice (20 months old) were randomly assigned to Control and *A. turkestanica* extract (50 mg/kg/day) groups. Duration: 28 days.	*Ajuga turkestanica* extract supplementation in aged mice increases Notch and Wnt signaling in triceps brachii muscle.	[226]
Total ecdysteroid extract from *A. turkestanica* (including 22-acetylcyasterone, cyasterone, ecdysterone, and turkesterone) and to assess the hypoglycemic activity of this extract on the model of alloxan-induced hyperglycemia and diabetes.	White mongrel male rats. The hyperglycemia model was induced by alloxan (150 mg/kg, s.c.). The PE preparation was introduced once per day in a dose of 5 mg/kg over a period of seven days.	Phytoecdysteroids extracted from *A. turkestanica* may be considered a promising hypoglycemic preparation.	[227]
Screen of *A. turkestanica* extract, rich in ecdysteroids for efficacy and safety.	C2C12 mouse myotube cell line.	The extract showed no androgenic activity within the dose range used. The potential for an *A. turkestanica* to support muscle mass without androgenic side effects.	[29]

## Data Availability

Not applicable.
